# Child maltreatment and hypertension in young adulthood

**DOI:** 10.1186/1471-2458-14-1149

**Published:** 2014-11-06

**Authors:** Shakira F Suglia, Cari J Clark, Renée Boynton-Jarrett, Nancy R Kressin, Karestan C Koenen

**Affiliations:** Department of Epidemiology, Mailman School of Public Health, Columbia University, 722 West 168th St, New York, NY 10032 USA; Department of Medicine, University of Minnesota Medical School, Minneapolis, MN USA; Department of Pediatrics, Boston University School of Medicine, Boston, MA USA; Senior Veterans Affairs Health Services Research and Development Research Career Scientist, VA Boston Healthcare System, Boston, MA 02130 USA; Medicine, Section of General Internal Medicine, Boston University School of Medicine, Boston, MA USA

**Keywords:** Child maltreatment, Hypertension, Violence, Stress, Blood pressure

## Abstract

**Background:**

Maltreatment during childhood and adolescence has been associated with chronic conditions in adulthood including cardiovascular disease. However, less is known about the effects of childhood maltreatment on cardiovascular risk factors prior to development of cardiovascular disease, or whether these effects are evident in young adulthood. Furthermore, few studies have examined sex differences and most studies have relied on self-reported outcome measures that are subject to misclassification.

**Methods:**

We examined the relationship between child maltreatment and hypertension in young adulthood in the National Longitudinal Study of Adolescent Health, a nationally representative school-based sample of US adolescents. Participants retrospectively (mean age 29.9, n = 11384) reported on their experiences of child maltreatment prior to the 6^th^ grade (prior to age 11) during follow-up. Child neglect, physical and sexual violence as well as a measure of social services visits to the home were examined. Blood pressure was measured during an in-home visit. Hypertension was defined as measured SBP of at least 140 mmHg or DBP of at least 90 mmHG measured in adulthood, or self-reported use of antihypertensive medications.

**Results:**

In adjusted models, women who experienced sexual abuse in early childhood had a higher prevalence of hypertension (Prevalence Ratio (PR) 1.43 95% CI 1.00, 2.05) compared to women who did not experience sexual abuse. Among men, experiencing sexual abuse was not statistically significantly associated with hypertension. Experiencing neglect, physical abuse or having visitations by social services at home during childhood was not associated with hypertension among either women or men.

**Conclusion:**

Sexual abuse in early childhood is associated with hypertension in young women.

## Background

Childhood maltreatment is a toxic stressor prevalent in the United States (US) [1]. The US state and local child protective services (CPS) estimate that 686,000 children were victims of some form of maltreatment in 2012 [2]. Recent data from the Behavioral Risk Factor Surveillance System (BRFSS) shows that 15% of adults report experiencing physical maltreatment during childhood. Maltreatment during childhood and adolescence has been associated with chronic conditions in adulthood including cardiovascular disease [1, 3, 4]. However, less is known about the effects of childhood maltreatment on cardiovascular risk factors prior to development of cardiovascular disease, or whether these effects are evident in young adulthood.

Only a handful of studies have explored the effects of childhood maltreatment and blood pressure in adulthood [5–8]. Using the Nurses Health Study II data, Riley and colleagues noted an association between severe and frequent experiences of child maltreatment and self-reported hypertension [7]. An analysis using the World Mental Health Survey demonstrated that experiencing physical abuse during childhood is associated with self-reported hypertension in adulthood [9]. In contrast, a study conducted among young adults in the US noted no significant association between child maltreatment and self-reported hypertension [8]. Other studies have noted significant associations only among women experiencing sexual abuse and not with other forms of child maltreatment [6].

Methodological limitations of the extant literature contribute to the discrepancy in study results and make it challenging to draw definitive conclusions. First, the vast majority of studies have relied on self-reported hypertension [7–9]. NHANES data has demonstrated that only 83% of hypertensive adults over age 18 are aware of their hypertension [10], thus, use of self-reported hypertension would underestimate the true prevalence of hypertension and potentially bias results toward the null. Second, most studies have not examined sex differences. Childhood maltreatment has been shown to differ between boys and girls, with girls more likely to experience sexual abuse compared to boys [11]. Furthermore, hypertension is more prevalent in men. Men are also more likely to be unaware of their blood pressure status but less likely to be taking antihypertensive medication [12], highlighting the importance of relying on measured blood pressure status to minimize misclassification. Third, differential behavioral responses to stress or physiological mechanisms may contribute to potential sex differences. Previous research has documented that, compared to women, men may be more likely to cope with stress by engaging in behaviors (i.e., smoking, alcohol consumption) which may contribute to elevated blood pressure [13]. Furthermore physiological responses to stress such as HPA axis dysregulation and inflammation have been noted to differ between sexes [14–17]. Thus, it may be important to examine sex differences in the relation between child maltreatment and hypertension.

In this study we examined child maltreatment and hypertension within the National Longitudinal Study of Adolescent Health (Add Health) a nationally representative longitudinal cohort of adolescents followed through young adulthood. Hypertension in young adulthood is a known risk factor for cardiovascular disease in adulthood. We focus on maltreatment that occurred early in childhood, prior to age 11. We improve upon other studies by using measured blood pressure to define hypertension, examining whether sex differences exist on the relationship between child maltreatment and hypertension, and examining the issue in a nationally representative sample.

## Methods

### Study population

The Add Health study is a nationally representative school-based, longitudinal study of the health-related behaviors of adolescents and their outcomes in young adulthood. An in-school questionnaire was administered to a nationally representative sample of students in grades 7 through 12, plus selected oversampled minority groups, stratified by age and sex, during the 1994–1995 school year in 132 schools. Four waves of in-home interviews (Wave 1, 1994–1995; Wave 2, 1996; Wave 3, 2001–2002; Wave 4, 2007–2008) were conducted. The study design has been described in detail elsewhere [18]. Briefly, 80 high schools representative of US schools were selected with respect to region of country, urbanicity, size, type and ethnicity. Eligible schools included an 11^th^ grade and enrolled more than 30 students. The first wave of in-home interviews was conducted between 1994 and 1995 (Wave 1), and approximately 20,745 adolescents, between the ages of 12 and 18 completed the in-home questionnaire at that time. Follow-up interviews for waves 3 (2001–02; response rate 77.4%) and 4 (2008–09; response rate 80.3%) were conducted with individuals who had participated in wave 1, leaving 12,288 participants who had sampling weight information. In the current analyses, those who were pregnant during wave 4 or who were missing blood pressure data were excluded, as well as those missing information on socio-demographics and covariates of interest leaving 11,384 as the final sample for our analyses. Participants who were included in the final sample differ from the full sample based on race/ethnicity. We used longitudinal sampling weights, available for participants followed to Wave 4, which adjust the sample to be representative of sample characteristics at baseline. The Add Health study was approved by the institutional review board of the University of North Carolina, Chapel Hill. The institutional review board of Columbia University, New York approved these analyses.

### Blood pressure assessment

During the fourth wave of follow-up (2007–2008, mean age 29) an in-home assessment was conducted and three blood pressure measurements were obtained with a Micro Life automated blood pressure monitor after the participant was seated for five minutes. The average of the last two measurements were used to calculate the average systolic blood pressure (SBP) and diastolic blood pressure (DBP). Participants were asked to provide to the interviewer all medications they were currently taking or had taken in the previous six months. Antihypertensive medications (beta-adrenergic blockers, calcium channel blockers, angiotensin converting enzymes) were identified from all medications provided. Hypertension was defined as having an elevated systolic blood pressure > = 140 mmHg or diastolic > = 90 mmHg (N = 2121) or reported using antihypertensive medications (N = 379).

### Child maltreatment

During the 3^rd^ wave of follow-up participants (Mean age 21.0) were retrospectively asked whether they had been the victims of abuse or neglect by their parent or caretaker when they were children by the time they started 6^th^ grade (approximately 11 years of age). Participants were also asked “How often had your parents or other adult care-givers slapped, hit, or kicked you?” (range 0 = never to 5 = more than ten times) and “How often had one of your parents or adult care-givers touched you in a sexual way, forced you to touch him or her in a sexual way, or forced you to have sexual relations?” (each item ranges from 0 = never happened to 5 = more than ten times). The frequency of some of the outcomes was too small to characterize as occurring more than once, for example the frequency of sexual abuse occurring more than once was 1.5%, hence physical abuse was defined as endorsing one time or more to the first question and sexual abuse as endorsing one or more times to the second question. Participants were asked “How often had your parents or adult caregivers not taken care of your basic needs such as keeping you clean or providing food or clothing”, (range 0 = never to 5 = more than ten times). Child neglect was defined as occurring if participants reported this occurred at least once. Furthermore, participants were asked prior to them entering the 6^th^ grade “How often had social services investigated how you were taken care of or tried to take you out of your living situation?”. Involvement with social services was defined as endorsing once or more times to this question.

### Covariates

Questionnaires ascertained information on socio-demographic factors, including age, sex, race/ethnicity and highest education level achieved. Highest education level was categorized as: less than high school, high school graduate, some college, college graduate or graduate education. Health behaviors were also assessed through questionnaire and were dichotomized into clinically meaningful categories. Current daily smoking in adulthood was defined as smoking at least one cigarette per day in the past 30 days. Physical activity was defined as 5 or more bouts of moderate to vigorous physical activity per week, based on self-report at the 4^th^ wave of follow-up. Alcohol use was dichotomized as consuming two drinks or less, or consuming more than two drinks at one sitting for men and among women, as consuming one drink or less, or consuming more than one drink per sitting. Participants also completed the Center for Epidemiologic Studies Depression Scale (CESD) -10 scale to assess depressive symptomatology [19]. High depression symptomatology was characterized as a score greater than 11 according to established guidelines [20]. During the fourth wave of follow-up visits, the participant’s height and weight were assessed. Obesity was defined as BMI > =30.

### Data analyses

Bivariate analyses examined the relation between child maltreatment and social services visits and hypertension among men and women. Given the high prevalence of hypertension in this sample, (28% men and 14% women) the use of odds ratios would be inappropriate [21], thus, binomial regression models were conducted to examine the relationship between child maltreatment and hypertension, adjusting for sociodemographic factors (age, race/ethnicity and highest education level attained), stratified by sex. Binomial regression models directly compute prevalence ratios (PR). The correlation between the types of maltreatment ranged from 0.12 to 0.30, thus they were jointly included in regression models. Thus, the PR for the association between each type of abuse and hypertension is adjusted for all other types. Three sets of models were conducted, both stratified by sex: 1) Adjusting for sociodemographic factors in model 1; 2) Further adjusting for the health behaviors of obesity, alcohol use, physical activity and smoking status at wave 4; and 2) Adjusting for covariates in models 1 and 2 and further adjusting for high depressive symptomatology. All analyses were weighted to account for the sampling method and conducted in SAS version 9.0 (SAS Institute, Cary, NC).

## Results

Table 1 shows the distribution of child maltreatment, socio-demographics, as well as study covariates. The sample comprises mostly of White (66%) men and women who were 29 years of age on average (SD 1.7, Range 25–34) at the time of the Wave 4 (2007–2008) assessment (Table 1). Twenty-eight percent of men had high blood pressure, as did 14% of women (Table 1). Cardiovascular risk factors were highly present in this sample, 36% of men and 38% of women were obese, and 27% of men and 22% of women were current smokers. Child maltreatment was also common, 14% of men and 8% of women reported ever experiencing neglect, 28% of men and 27% women reported experiencing physical maltreatment and 5% of both men and women reported experiencing sexual abuse as a child. In addition 4% of men and 5% of women reported their family being investigated by social services.

**Figure 1:**
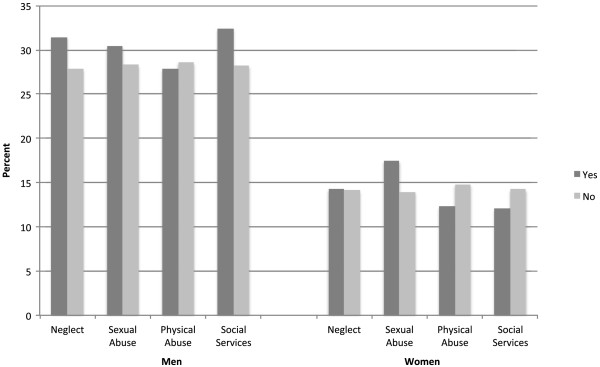
**Weighted prevalence of hypertension by type of child maltreatment and involvement with social services stratified by sex, Add Health Study N = 11384.** Hypertension was defined as having an elevated systolic blood pressure > = 140 mmHg or diastolic > = 90 mmHg or reported using antihypertensive medications. ^1^No statistically significant differences were noted between type of child maltreatment and hypertension.

The prevalence of hypertension by child maltreatment status and social services visitation is presented in Figure 1. Men who experienced neglect (31% vs. 28%), sexual abuse (30% vs. 28%) or whose families were investigated by social services (32% vs. 28%) had a higher prevalence of hypertension compared to those who did not endorse those experiences, however these were not statistically significant differences. Women who experienced sexual abuse had a non-statistically significant higher prevalence of hypertension (17% vs. 14%) compared to those who did not. In contrast those who experienced physical abuse (12% vs. 15%) or whose families were involved with social services (12% vs. 14%) had a lower prevalence of hypertension compared to those who did not endorse those experiences, however these differences were not statistically significant.

**Table 1:** **Weighted prevalence of study sample demographics, covariates, child maltreatment and blood pressure status, Add Health study N = 11384**

	Men (N =5355)	Women (N =6029)	
	Mean/% (SE)	Mean/% (SE)	p-value
**Age (Mean)**	29.01 (0.04)	28.86 (0.03)	0.0040
**Race/Ethnicity**			0.1789
White	66.00 (0.88)	66.34 (0.80)	
African American	14.79 (0.65)	16.16 (0.57)	
Hispanic	12.20 (0.60)	11.36 (0.58)	
Other race/ethnicity	7.01 (0.45)	6.14 (0.39)	
**Highest education level**			< 0.0001
Less than high School	9.73 (0.58)	6.96 (0.48)	
High School graduate	24.46 (0.84)	16.87 (0.67)	
Some college	37.84 (0.90)	41.41 (0.86)	
College or graduate education	27.97 (0.83)	34.76 (0.82)	
**Depressive symptoms**	13.53 (0.67)	19.26 (0.71)	<0.0001
**Hypertension** ^**1**^	28.39 (0.85)	14.14 (0.60)	<0.0001
**Physical activity** ^**2**^	57.16 (0.93)	50.26 (0.87)	<0.0001
**Current daily smoking** ^**3**^	26.56 (0.47)	22.37 (0.38)	0.0002
**Obesity (BMI >30)**	35.81 (0.90)	38.03 (0.85)	0.070
**Alcohol use** ^**4**^	54.01 (0.94)	57.78 (0.86)	0.0030
**Child maltreatment**			
Neglect	14.02 (0.67)	8.41 (0.47)	<0.0001
Physical	28.33 (0.85)	26.75 (0.76)	0.1635
Sexual	4.53 (0.41)	4.73(0.36)	0.7242
**Social service investigation of the family**	4.00 (0.37)	4.68 (0.36)	0.1881

^1^Hypertension was defined as having an elevated systolic blood pressure > = 140 mmHg or diastolic > = 90 mmHg or reported using antihypertensive medications.

^2^Physical activity defined as 5 or more bouts of moderate to rigorous physical activity per week.

^3^Current daily smoking defined as smoking at least one cigarette per day in the past 30 days.

^4^Alcohol use defined consuming more than two drinks at one sitting for men and among women, as consuming more than one drink per sitting.

In models adjusted for socio-demographic factors, and other forms of maltreatment, women who experienced sexual abuse in early childhood had a significantly higher prevalence of hypertension (PR 1.43 95% CI 1.00, 2.05) compared to women who did not experience sexual abuse (Table 2). Among men, experiencing sexual abuse in early childhood was not associated with higher prevalence of hypertension (PR 0.98 95% CI 0.72, 1.32). Experiencing neglect, physical abuse or having visitations by social services at home during childhood was not associated with hypertension among either women or men. While the effect estimates did not substantially change upon inclusion of other covariates, their statistical significance did change. After adjustment for physical activity, current smoking, obesity, alcohol use and depressive symptomatology, women experiencing sexual abuse had a higher prevalence of hypertension (PR 1.35 95% CI 0.92, 1.96) albeit this was no longer statistically significant.

**Table 2:** **Child maltreatment prior to entering the 6** ^**th**^ **grade (11 years of age) and hypertension, stratified by sex Add Health study N = 11384**

	Hypertension model 1		Hypertension model 2		Hypertension model 3	
	Men	Women	Men	Women	Men	Women
	PR (95% CI)	PR (95% CI)	PR (95% CI)	PR (95% CI)	PR (95% CI)	PR (95% CI)
**Child maltreatment**						
**Neglect**	1.12 (0.95, 1.32)	0.93 (0.70, 1.22)	1.11 (0.94, 1.30)	1.00 (0.74, 1.33)	1.11 (0.94, 1.30)	1.00 (0.74, 1.34)
**Physical abuse**	0.93 (0.81, 1.08)	0.82 (0.65, 1.02)	0.93 (0.80, 1.08)	0.81 (0.64, 1.01)	0.93 (0.80, 1.08)	0.81 (0.64, 1.01)
**Sexual abuse**	0.98 (0.72, 1.32)	1.43 (1.00, 2.05)*	0.94 (0.69, 1.29)	1.35 (0.92, 1.96)	0.94 (0.69, 1.29)	1.35 (0.92, 1.96)
**Social services**	1.13 (0.85, 1.51)	0.79 (0.52, 1.22)	1.17 (0.87, 1.58)	0.78 (0.50, 1.22)	1.18 (0.88, 1.58)	0.78 (0.50, 1.22)
**Obesity**	----	------	1.92 (1.69, 2.19)*	2.33 (1.94, 2.81)*	1.92 (1.69, 2.18)*	2.33 (1.94, 2.81)*
**Current smoking**	----	------	1.05 (0.91, 1.21)	1.13 (0.89, 1.44)	1.05 (0.91, 1.22)	1.13 (0.89, 1.44)
**Alcohol use**	----	------	1.02 (0.90, 1.15)	0.96 (0.80, 1.16)	1.01 (0.89, 1.15)	0.96 (0.80, 1.16)
**Physical activity**	----	------	1.04 (0.91, 1.18)	0.82 (0.70, 0.97)	1.04 (0.91, 1.18)	0.82 (0.70, 0.97)
**Depression symptoms**	----	------	----	------	0.92 (0.75, 1.13)	0.99 (0.80, 1.23)

All models adjusted for age, race/ethnicity and education level.

*p <0.05.

## Discussion

In this study we found evidence of an association between child sexual abuse and hypertension among women but noted no associations among other forms of maltreatment and hypertension or among any of the forms of child maltreatment examined and hypertension among men. A positive relation between child maltreatment and hypertension in adulthood has been previously demonstrated [6–9, 22]. For example, Riley and colleagues, using the Nurses Health Study II data, were able to characterize abuse that occurred prior to age 11 and later experiences of abuse noting that those who experience abuse in both childhood and adolescence, classified as more severe and frequent, had a higher risk of self-reported hypertension [7]. Our results, using actual blood pressure data, validate and extend these prior results using self-reported data.

Sex differences in the association between maltreatment and hypertension have, to our knowledge, only been examined in one other study; an analysis using the National Comorbidity Survey showed that experiencing physical abuse during childhood is associated with self-reported hypertension among adult men but not women, and sexual abuse was associated with self-reported hypertension among women but not men [6]. Prior studies have highlighted sex-specific responses in blood pressure to job strain [23–25] with negative consequences for men. In contrast we note no relation between physical abuse and hypertension among men or women but note a significant association between sexual abuse and hypertension among women. Differential coping mechanisms or differential HPA axis dysregulation in response to stress between men and women may account for the differences noted [14–17, 26].

To our knowledge, all studies examining child maltreatment and hypertension in adulthood, which have noted significant associations, have used self-reported hypertension [6, 7, 9, 22, 27]. In contrast we use measured blood pressure to define hypertension thus reducing the potential for self-report bias. A recent study of the relation between intimate partner violence and cardiovascular endpoints in the Nurses Health Study, noted stronger associations between non-verified cardiovascular outcomes and violence exposure compared to associations that relied on verified cardiovascular outcomes suggesting self-report bias [28].

The effects of chronic stressors, such as violence, on hypertension can be the result of modified health behaviors known to be affected by chronic stressors; smoking, drinking, and poor dietary habits have been shown to be more prevalent in stressful environments and have also been shown to be associated with cardiovascular disease, including hypertension. Adjusting for obesity, physical activity, smoking and alcohol consumption in our analyses did not change the effect estimates noted though the effect of sexual abuse on hypertension among women was no longer statistically significant. A more direct pathway through which stress can affect health is through its activation of the autonomic nervous system. Animal studies have demonstrated that hypertension following social stress is associated with increased norepinephrine turnover, with other mechanistic studies providing evidence for the role of inflammatory responses and renal mechanisms [29]. Chronic stress has also been shown to induce a chronic and systemic state of mild inflammation (i.e., C-reactive protein and interleukin-6) a key mechanism in the development of cardiovascular disease [30].

There are a number of limitations worth mentioning. First, as is typical with longitudinal studies, there is a reduction in the sample available from the original Add Health cohort over time; it is likely the loss to follow-up is non-differential with respect to outcome, potentially biasing our results towards the null. However, we use longitudinal sampling weights, which adjust the sample to be representative of sample characteristics at baseline. Second, while the retrospective assessment of maltreatment was collected prior to our outcome assessment it consisted of only three questions that did not account for the severity of the exposures. Thus we were limited to a yes/no characterization of maltreatment exposure. A more detailed assessment that would query about different experiences of neglect, emotional, sexual and physical abuse separately would allow for better classification of severity of exposure. However, the fact that we were able to detect associations between these crude measures of maltreatment and hypertension suggest the dynamic is robust. Third, our assessment of blood pressure was conducted at only one point in time; multiple assessments of blood pressure would provide a more reliable measure of hypertension in this sample. However, we improve upon other studies that relied solely on self-reported hypertension. Finally, as noted in prior studies, the prevalence of hypertension in this national sample of young adults is higher than in other nationally representative samples [31, 32]. If the prevalence of hypertension is associated with inaccurate measurement then this would have likely led to underestimation of the association between maltreatment and hypertension in our analysis.

## Conclusion

This study is one of the first analyses of the effects of child maltreatment on measured blood pressure, noting a significant relation between child sexual abuse and hypertension among women that may be partially attributed to health behaviors. Detection of child abuse early in the life course, coupled with appropriate psychological counseling and health behavior management may prevent the development of hypertension among women along with numerous adverse health conditions. Given the high prevalence of hypertension in this national sample of young adults [31, 32], a focus on the identification of risk factors early in the life course is warranted. Future studies that examine how child maltreatment impacts the trajectory of health behaviors and health outcomes across the life course can help identify mechanisms and modifiable factors that can be used in intervention and prevention efforts.

## References

[1] Shonkoff JP, Garner AS (2012). The lifelong effects of early childhood adversity and toxic stress. Pediatrics.

[2] Child Maltreatment Facts at a Glance 2014http://www.cdc.gov/violenceprevention/pdf/childmaltreatment-facts-at-a-glance.pdf

[3] Felitti VJ, Anda RF, Nordenberg D, Williamson DF, Spitz AM, Edwards V, Koss MP, Marks JS (1998). Relationship of childhood abuse and household dysfunction to many of the leading causes of death in adults: the Adverse Childhood Experiences (ACE) study. Am J Prev Med.

[4] Rich-Edwards JW, Mason S, Rexrode K, Spiegelman D, Hibert E, Kawachi I, Jun HJ, Wright RJ (2012). Physical and sexual abuse in childhood as predictors of early-onset cardiovascular events in women. Circulation.

[5] Azar S, Malwah BM (2002). Parenting and child maltreatment. Handbook of Parenting: Vol 4 Social conditions and applied parenting.

[6] Goodwin RD, Stein MB (2004). Association between childhood trauma and physical disorders among adults in the United States. Psychol Med.

[7] Riley EH, Wright RJ, Jun HJ, Hibert EN, Rich-Edwards JW (2010). Hypertension in adult survivors of child abuse: observations from the Nurses’ Health Study II. J Epidemiol Community Health.

[8] Nomura Y, Chemtob CM (2007). Conjoined effects of low birth weight and childhood abuse on adaptation and well-being in adolescence and adulthood. Arch Pediatr Adolesc Med.

[9] Stein DJ (2009). Early childhood adversity and later hypertension: data from the World Mental Health Survey. Ann Clin Psychiatry.

[10] Nwankwo T, Yoon SS, Burt V, Gu Q (2013). Hypertension among adults in the United States: National Health and Nutrition Examination Survey, 2011–2012. NCHS Data Brief.

[11] Finkelhor D, Turner HA, Shattuck A, Hamby SL (2013). Violence, crime, and abuse exposure in a national sample of children and youth: an update. JAMA Pediatrics.

[12] Yoon SS, Burt V, Louis T, Carroll MD: **Hypertension among adults in the United States, 2009–2010.** *NCHS Data Brief* 2012, (107)**:**1–8. 23102115

[13] Lindquist TL, Beilin LJ, Knuiman MW (1997). Influence of lifestyle, coping, and job stress on blood pressure in men and women. Hypertension.

[14] Thorand B, Baumert J, Kolb H, Meisinger C, Chambless L, Koenig W, Herder C (2007). Sex differences in the prediction of type 2 diabetes by inflammatory markers: results from the MONICA/KORA Augsburg case-cohort study, 1984–2002. Diabetes Care.

[15] Rohleder N, Schommer NC, Hellhammer DH, Engel R, Kirschbaum C (2001). Sex differences in glucocorticoid sensitivity of proinflammatory cytokine production after psychosocial stress. Psychosom Med.

[16] Darnall BD, Suarez EC (2009). Sex and gender in psychoneuroimmunology research: past, present and future. Brain Behav Immun.

[17] Kajantie E, Phillips DI (2006). The effects of sex and hormonal status on the physiological response to acute psychosocial stress. Psychoneuroendocrinology.

[18] Popkin BM, Udry JR (1998). Adolescent obesity increases significantly in second and third generation U.S. immigrants: the National Longitudinal Study of Adolescent Health. J Nutr.

[19] Radloff LS (1991). The use of the Center for Epidemiologic Studies Depression Scale in adolescents and young adults. J Youth Adolesc.

[20] **Center for Epidemiological Studies Depression Scale (CES-D)** http://www.scireproject.com/outcome-measures-new/centerepidemiological-studies-depression-scale-ces-d-and-ces-d-10

[21] Greenland S (2004). Model-based estimation of relative risks and other epidemiologic measures in studies of common outcomes and in case–control studies. Am J Epidemiol.

[22] Springer KW, Sheridan J, Kuo D, Carnes M (2007). Long-term physical and mental health consequences of childhood physical abuse: results from a large population-based sample of men and women. Child Abuse Negl.

[23] Sparrenberger F, Cichelero FT, Ascoli AM, Fonseca FP, Weiss G, Berwanger O, Fuchs SC, Moreira LB, Fuchs FD (2009). Does psychosocial stress cause hypertension? a systematic review of observational studies. J Hum Hypertens.

[24] Cesana G, Sega R, Ferrario M, Chiodini P, Corrao G, Mancia G (2003). Job strain and blood pressure in employed men and women: a pooled analysis of four northern italian population samples. Psychosom Med.

[25] Ohlin B, Berglund G, Rosvall M, Nilsson PM (2007). Job strain in men, but not in women, predicts a significant rise in blood pressure after 6.5 years of follow-up. J Hypertens.

[26] Hanninen V, Aro H (1996). Sex differences in coping and depression among young adults. Soc Sci Med.

[27] Afifi TO, Mota N, MacMillan HL, Sareen J (2013). Harsh physical punishment in childhood and adult physical health. Pediatrics.

[28] Mason SM, Wright RJ, Hibert EN, Spiegelman D, Jun HJ, Hu FB, Rich-Edwards JW (2012). Intimate partner violence and incidence of type 2 diabetes in women. Diabetes Care.

[29] Steptoe A, Kivimaki M (2013). Stress and cardiovascular disease: an update on current knowledge. Annu Rev Public Health.

[30] Ranjit N, Diez-Roux AV, Shea S, Cushman M, Seeman T, Jackson SA, Ni H (2007). Psychosocial factors and inflammation in the multi-ethnic study of atherosclerosis. Arch Intern Med.

[31] Nguyen QC, Tabor JW, Entzel PP, Lau Y, Suchindran C, Hussey JM, Halpern CT, Harris KM, Whitsel EA (2011). Discordance in national estimates of hypertension among young adults. Epidemiology.

[32] Chyu L, McDade TW, Adam EK (2011). Measured blood pressure and hypertension among young adults: a comparison between two nationally representative samples. Biodemography Soc Biol.

[CR33] The pre-publication history for this paper can be accessed here:http://www.biomedcentral.com/1471-2458/14/1149/prepub

